# Spatial Deconvolution Method for the Diagnosis of Conformal Array Antennas

**DOI:** 10.3390/s26216819

**Published:** 2026-10-28

**Authors:** Yuxin Ren, Junhao Zheng, Xiaobo Liu, Guan-Long Huang, Xiaoming Chen, Ahmed A. Kishk

**Affiliations:** 1 School of Information and Communication Engineering, Xi’an Jiaotong University, Xi’an 710049, China; renyuxin@caict.ac.cn (Y.R.); xiaoboliu@xjtu.edu.cn (X.L.); 2 College of Communication and Information Technology, Xi’an University of Science and Technology, Xi’an 710054, China; 1071218679@stu.xjtu.edu.cn; 3 School of Electronic and Information Engineering, Foshan University, Foshan 528225, China; hgl@fosu.edu.cn; 4 Department of Electrical and Computer Engineering, Concordia University, Montreal, QC H3G 1M8, Canada; kishk@encs.concordia.ca

**Keywords:** spherical spatial deconvolution, conformal array antenna, fault element diagnosis, field inversion

## Abstract

The paper presents an effective fault-element detection method for conformal array antennas based on a spherical spatial deconvolution. To address the diagnosis issue caused by curved array surfaces, the proposed method extends two-dimensional planar spatial deconvolution to three-dimensional spherical spatial deconvolution via spherical integral transformation, making it well suited for spherical conformal array antennas. Since spatial convolution–deconvolution mathematically corresponds to multiplication and division operations in the wavenumber domain, spherical deconvolution can be readily implemented by combining matrix division and inverse fast Fourier transform. Notably, the size of the spatial deconvolution kernel is a critical parameter that affects the reconstruction accuracy and computational overhead of the entire diagnostic procedure. Numerical simulation and measurement results show that the proposed method achieves better accuracy in field inversion than the conventional spherical wave expansion method.

## 1. Introduction

An anechoic chamber (AC) is commonly used for measuring the near fields of the antenna under test (AUT). It uses a near-to-far-field transformation (NFT) algorithm to extrapolate the far-field pattern; the spherical near-field measurement system is widely used because of its small truncation error and high efficiency in multi-probe anechoic chambers [1,2,3]. The conventional spherical near-to-far-field transformation (SNFT) solves outwardly along the radial direction of the measured sphere and can effectively characterize far-field radiation properties. However, in some application scenarios, testing is required not only for the far-field performance but also for the amplitude-phase distribution characteristics near the AUT aperture (usually lies in the reactive near-field region), which can intuitively embody the operational performance of the AUT and is adopted for antenna diagnostic purposes [4,5,6]. Specifically, the diagnostic capability is particularly valuable for electrically large conformal antennas, whose surfaces are mounted by numerous radiating elements, whereas individual elements may degrade in performance or fail due to material aging, assembly errors, and fabrication imperfections. In such cases, acquiring accurate reactive-near-field data is essential for comprehensive fault inspection across the entire conformal antenna aperture [7]. In this regard, accurately obtaining the aperture near field near the reactive region is an important research subject.

Currently, several effective approaches for backward aperture-field reconstruction have been reported. The source reconstruction method (SRM) characterizes the aperture near-field current via integral equations and adopts a regularization technique to improve the convergence of the iterative solution [8,9,10,11]. Such inverse studies generally deliver enhanced regularization performance at the cost of computational complexity. Moreover, the objectives were sometimes ill-posed, and optimal solutions could not be obtained. The iterative aperture field reconstruction method in the spatial-wavenumber domain under Cartesian coordinates was fully studied in [12,13,14], where the planar inverse Fourier transform (IFT) was used to reconstruct the aperture near field with a lower truncation error. In addition, the spatial deconvolution method was proposed to invert the reactive near-field; its accuracy and time cost depend on the deconvolution kernel matrix size in the wavenumber domain [15,16,17]. In other words, the spatial deconvolution can reduce the truncation error by enlarging the deconvolution kernel [18]. More importantly, probe compensation essentially performs deconvolution between the radiation pattern of the AUT and that of the probe, and this operation can be implemented more conveniently via spatial deconvolution. Meanwhile, compared with the IFT method, the deconvolution method is more readily applied in cylindrical and spherical coordinate systems when the sampling interval exceeds the Nyquist sampling interval.

To enable inverse aperture near-field derivation in different coordinate systems, some researchers have applied spatial- and wavenumber-domain transformation methods. Specifically, the spherical-wave-expansion (SWE) to plane-wave-expansion (PWE) antenna diagnostic technique was presented in [19,20,21,22], where the wave expansion coefficients were transformed in the wavenumber domain, and the inverse near-field projection method can be executed under the planar coordinates. This method offers a lower truncation error and computational complexity than the IFT and SRM methods [23]. Furthermore, the authors of [24] verified the effectiveness of the spatial deconvolution method based on the plane-to-sphere geometric projection in the spatial domain, establishing a concise and low-cost spherical reactive near-field reconstruction method. Nevertheless, this method was limited to the forward hemisphere and thus restricted its application to the entire spherical inward projection.

Unlike previous works, this paper presents a spherical deconvolution method in the full spatial domain to accurately obtain the reactive near field close to the conformal AUT aperture. Specifically, the method uses matrix division and fast Fourier transform (FFT) to perform spatial deconvolution in the wavenumber domain. The deconvolution kernel in the spatial domain determines the accuracy and the computational cost of the entire process. The simulation and measurement results demonstrate the proposed method’s accuracy superiority over the conventional spherical wave expansion method using the Hankel function of the first kind, although the proposed method has a larger time cost. It uniquely removes the forward-hemisphere restriction of existing spatial deconvolution schemes, supporting full-sphere inward projection and efficient reactive near-field reconstruction for conformal antennas.

## 2. Theory

In order to characterize the electric field in any outward spatial region, the vector potential should be known. Thus, this section first presents the scalar potential solution, then derives the vector potential in spherical coordinates, and then obtains the spatial convolution-deconvolution method.

Assume that the scalar electric potential Φ(r) under the spherical coordinate satisfies the Helmholtz equation

(1) $$\nabla^{2}\Phi (r)+k^{2}\Phi (r)=h(r)$$

where r=(r,θ,φ) is the coordinate of an arbitrary extrapolated sphere S, k=2πf/c is the wavenumber, f is the operating frequency, c is the lightspeed, and h(r) is the scalar source in free space. h(r) can be decomposed into a linear combination of basis functions, and the solution of h(r) can be expressed as

(2) $$h(r)=∭h(r^{\prime })\delta (r-r^{\prime }){r^{\prime }}^{2}\sin\theta^{\prime }dr^{\prime }d\theta^{\prime }d\varphi^{\prime }=\iint h(r^{\prime })\delta (r-r^{\prime })\sin\theta^{\prime }d\theta^{\prime }d\varphi^{\prime }$$

where δ is the Dirac delta function, and $r^{\prime }$ is the coordinate on the measured sphere $S^{\prime }$ enclosing the AUT (i.e., S is larger than $S^{\prime }$). Since the integral is performed along the surface of $S^{\prime }$, the volume integral can be reduced to a surface integral over the sphere $S^{\prime }$. Accordingly, the solution of Φ(r) can be calculated as

(3) $$\Phi (r)=\iint h(r^{\prime })g(r-r^{\prime })\sin\theta^{\prime }d\theta^{\prime }d\varphi^{\prime }$$

where g can be regarded as the impulse response of the linear system and satisfies the Helmholtz equation of Equation (1). The solution of g can be defined as the Green function and written as

(4) $$g(r-r^{\prime })=\frac{e^{-jk\left|r-r^{\prime }\right|}}{4\pi \left|r-r^{\prime }\right|}$$

According to the equivalence theorem, the aperture of the AUT is treated as a perfect electric conductor (PEC), and the image magnetic current and the original magnetic current superpose to double the equivalent magnetic current. So, the electric field enclosed in $S^{\prime }$ can be represented by the equivalent magnetic source $M_{1}(r_{1})$ on the surface of $S^{\prime }$:

(5) $$M_{1}(r^{\prime })=-2\hat{n}\times E_{1}(r^{\prime })$$

where $E_{1}(r^{\prime })$ is the measured spherical radiative near-field on $S^{\prime }$, and $\hat{n}$ is the outward unit normal vector to $S^{\prime }$. By substituting Equation (5) into Equation (3), the vector electric potential $F_{1}(r)$ can be obtained as

(6) $$\begin{array}{cc} F_{1}(r) & =\frac{1}{4\pi }\iint \frac{M_{1}(r^{\prime })e^{-jk\left|r-r^{\prime }\right|}}{\left|r-r^{\prime }\right|}\sin\theta^{\prime }d\theta^{\prime }d\varphi^{\prime }\\ & =\iint M_{1}(r^{\prime })G(r-r^{\prime })\sin\theta^{\prime }d\theta^{\prime }d\varphi^{\prime } \end{array}$$

When the vector potential $F_{1}(r)$ in the outward free space on an arbitrary sphere S is known, the spherical electric field on S can be calculated as

(7) $$E_{2}(r)=-j\omega F_{1}(r)-\nabla \phi$$

where the scalar potential in passive region can be calculated as

(8) $$\nabla \cdot F_{1}(r)+j\omega \mu \varepsilon \phi =0\Rightarrow \phi =-\frac{1}{j\omega \mu \varepsilon }\nabla \cdot F_{1}(r)$$

Substituting Equation (8) into Equation (7), $E_{2}(r)$ can be calculated as

(9) $$E_{2}(r)=-j\omega F_{1}(r)+\frac{1}{j\omega \mu \varepsilon }\nabla \left(\nabla \cdot F_{1}(r)\right)$$

where $k=\omega \sqrt{\mu \varepsilon }$. Then, the divergence of vector potential $F_{1}(r)$ under the spherical coordinate can be calculated as

(10) $$\nabla \cdot F_{1}=\frac{1}{r^{2}}\frac{\partial \left(r^{2}F_{1,r}\right)}{\partial r}+\frac{1}{r\sin\theta }\frac{\partial \left(F_{1,\theta }\sin\theta \right)}{\partial \theta }+\frac{1}{r\sin\theta }\frac{\partial \left(F_{1,\varphi }\right)}{\partial \varphi }$$

Thus, substituting Equation (10) into Equation (9), the three spherical coordinate components can be represented as

(11) $$E_{2,r}=-j\omega F_{1,r}-\frac{j\omega }{k^{2}}\frac{\partial }{\partial r}\left(\nabla \cdot F_{1}\right)$$

(12) $$E_{2,\theta }=-j\omega F_{1,\theta }-\frac{j\omega }{k^{2}}\frac{1}{r}\frac{\partial }{\partial \theta }\left(\nabla \cdot F_{1}\right)$$

(13) $$E_{2,\varphi }=-j\omega F_{1,\varphi }-\frac{j\omega }{k^{2}}\frac{1}{r\sin\theta }\frac{\partial }{\partial \varphi }\left(\nabla \cdot F_{1}\right)$$

Now, by substituting Equation (6) into Equations (11)–(13), the electric field on S can be expressed as

(14) $$\begin{array}{cc} E_{2}(r) & =-j\omega \iint M_{1}(r^{\prime })G(r-r^{\prime })\sin\theta^{\prime }d\theta^{\prime }d\varphi^{\prime }-\frac{j\omega }{k^{2}}\nabla \left[\nabla \cdot \iint M_{1}(r^{\prime })G(r-r^{\prime })\sin\theta^{\prime }d\theta^{\prime }d\varphi^{\prime }\right]\\ & =\iint -j\omega M_{1}(r^{\prime })G(r-r^{\prime })-\frac{j\omega }{k^{2}}\nabla \left[\nabla \cdot M_{1}(r^{\prime })G(r-r^{\prime })\right]\sin\theta^{\prime }d\theta^{\prime }d\varphi^{\prime } \end{array}$$

Define the impulse response under the spherical coordinate as

(15) $$H=-j\omega IG(r-r^{\prime })-\frac{j\omega }{k^{2}}\nabla \left[\nabla \cdot IG(r-r^{\prime })\right]$$

where I is the unit dyad. Since the sampling is performed on the sphere $S^{\prime }$, and the convolution process can be represented as θ and φ variables (i.e., the radius is constant and the convolution kernel H slides on the sphere), the spherical electric field of Equation (14) can be written as

(16) $$E_{2}(\theta ,\varphi )=\iint M_{1}(\theta^{\prime },\varphi^{\prime })H(\theta -\theta^{\prime },\varphi -\varphi^{\prime })\sin\theta^{\prime }d\theta^{\prime }d\varphi^{\prime }$$

Then, by combining Equations (5)–(15), Equation (16) can be simplified as

(17) $$E_{2}(\theta ,\varphi )=\iint_{S}E_{1}(\theta^{\prime },\varphi^{\prime })G(\theta -\theta^{\prime },\varphi -\varphi^{\prime })\sin\theta^{\prime }d\theta^{\prime }d\varphi^{\prime }$$

Notably, Equation (17) is the spatial convolution process; so, the corresponding matrix multiplication in the wavenumber domain can be expressed as

(18) $$E_{2}=F^{-1}\left[F\left[E_{1}\right]⊙F\left[G\right]\right]$$

where F and $F^{-1}$ are the forward and inverse Fourier transforms, and ⊙ is the Hadamard product. Now, the spherical electric field $E_{2}$ on an arbitrary outward sphere S can be derived from the radiative near field $E_{1}$ on the measured sphere $S^{\prime }$. Correspondingly, the spatial deconvolution can be realized by the inverse procedure of Equation (18) as

(19) $$E_{3}=F^{-1}\left[F\left[E_{1}\right]⊘F\left[G\right]\right]$$

where ⊘ is the element-wise division of two matrices in the wavenumber domain. Equation (19) is the desired spatial deconvolution method to obtain the reactive near field $E_{3}$ on an arbitrary inward sphere $S^{\prime \prime }$ inside $S^{\prime }$ by the measured near field $E_{1}$ on the sphere $S^{\prime }$.

In addition, the computational cost of the spatial deconvolution process is determined by the size of the deconvolution kernel G. Therefore, to better understand the implementation procedures of the proposed method, the visual theoretical illustrations are presented below.

**Figure 1:**
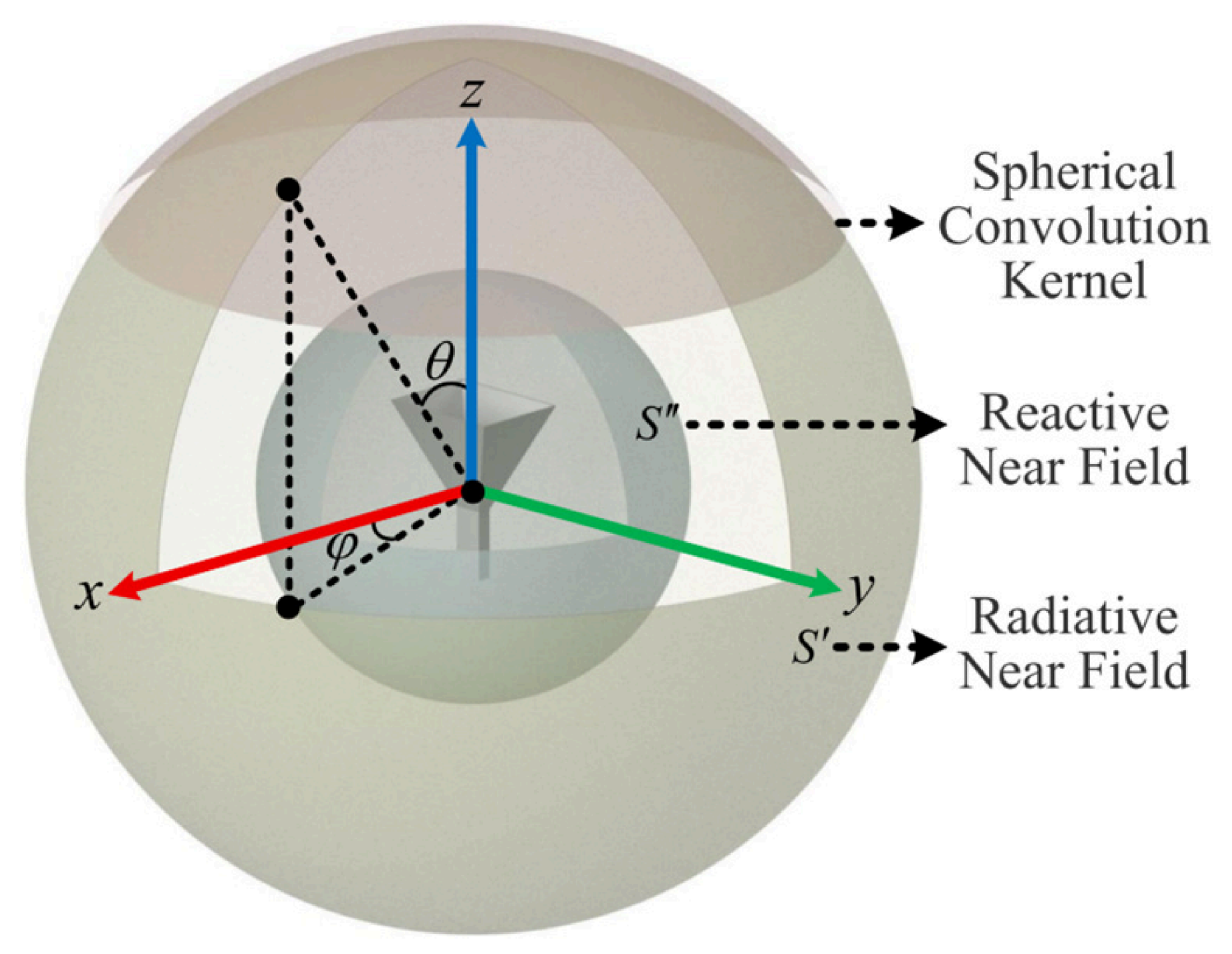
Geometric structure of the spatial deconvolution method.

**Figure 2:**
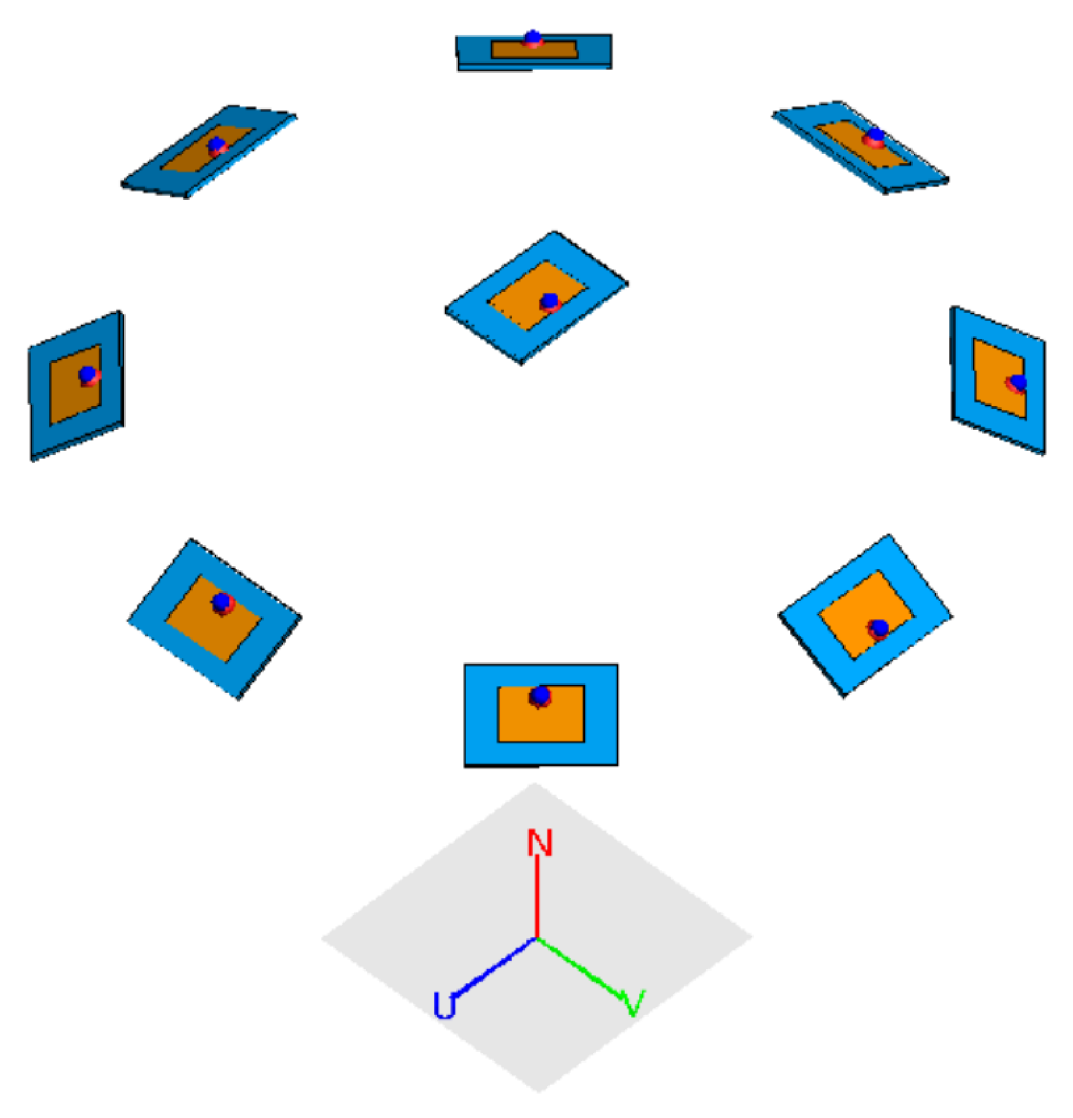
Simulation model of partial-spherical conformal AUT in FEKO.

The spatial deconvolution kernel G is defined in Figure 1, whose size is determined by the integral ranges along the θ and φ directions. The reactive near field on $S^{\prime \prime }$ is inversely retrieved from the radiative near field on $S^{\prime }$. Specifically, the proposed deconvolution procedures can be executed as follows:

(1) G is sampled in M and N points in θ and φ with the intervals of Δθ and Δφ. The shape of G is determined as a partial-spherical dome (red dome in Figure 1);
(2) Then, the partial-spherical dome is rotated along the θ direction at an initial $\varphi_{1}$ angle, where each position at $\left(\theta_{n},\varphi_{1}),\;(n=1,2,\ldots N\right)$ is calculated by Equation (19) to inversely retrieve the reactive near field on $S^{\prime \prime }$ from the radiative near field on $S^{\prime }$;
(3) When the entire ranges $\theta =(\theta_{1}\sim \theta_{N})$ at $\varphi_{1}$ are scanned, G moves to the next φ+Δφ angle, and G continues to scan over the full $\theta =(\theta_{1}\sim \theta_{N})$ range;
(4) The second and third steps are iteratively executed until the whole scanning of $\theta =(\theta_{1}\sim \theta_{N})$ and $\varphi =(\varphi_{1}\sim \varphi_{M})$ is finished (i.e., the total sphere $S^{\prime }$ is fully scanned);
(5) Finally, the complete reactive near field on $S^{\prime \prime }$ is obtained, which can be used to observe the field distribution and element diagnosis of the conformal AUT.

The computational complexity depends on the size of G and the scanning intervals Δθ and Δφ. Thus, a larger G and smaller Δθ and Δφ yield more accurate results but at a higher time cost. Notably, the proposed method can retrieve the reactive near field from a partial-sphere or full-sphere radiative near-field or far-field region, which will be verified in the following results.

## 3. Results and Discussion

### 3.1. Simulation Results

**Figure 3:**
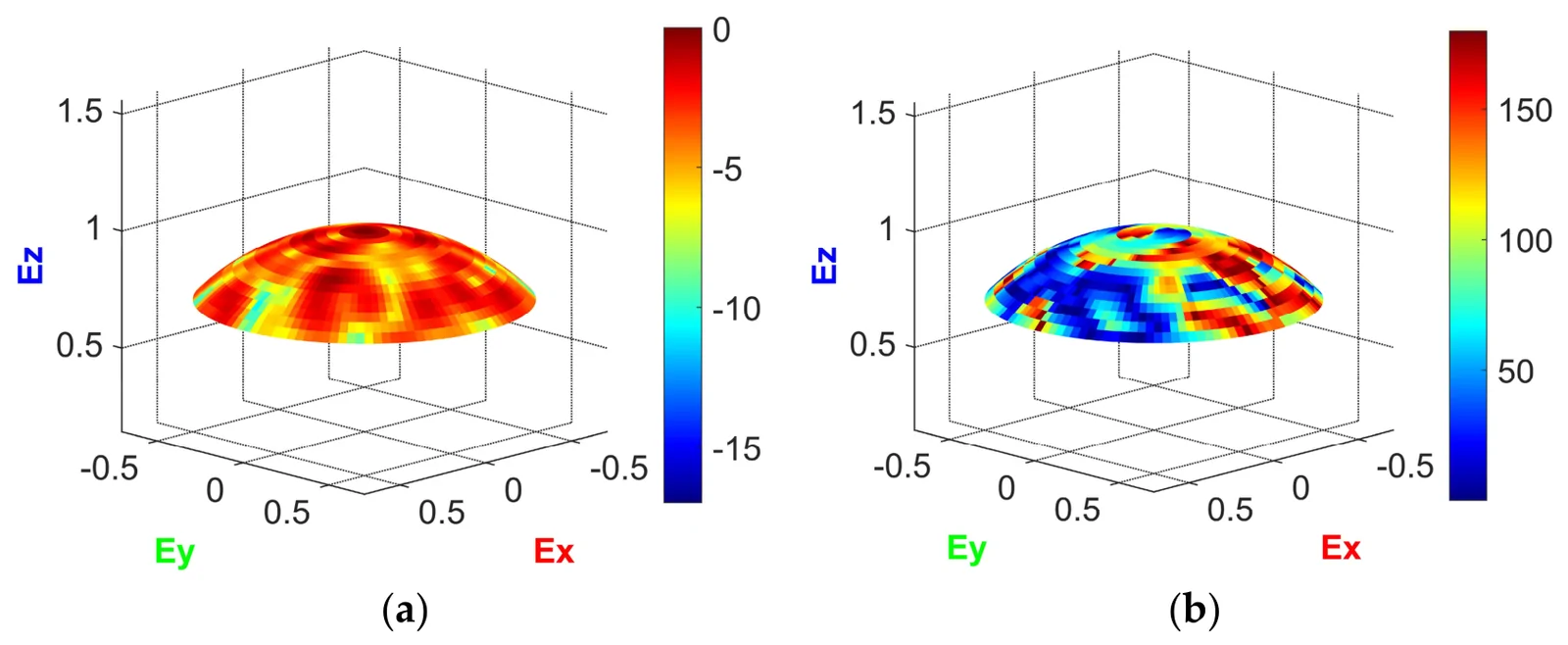
Simulated radiative near field on $S^{\prime }$: (**a**) amplitude and (**b**) phase.

The partial-spherical conformal AUT composed of nine patch elements working at 3 GHz is simulated in FEKO, as shown in Figure 2, where the radius *r*_0_ of the conformal structure is 5.07*λ*, the distance between two elements along the *θ* direction is 2.6*λ*, the radius *r*′ of the radiative near-field sphere $S^{\prime }$ is 50λ (the radiative near-field range is limited within $2D^{2}/\lambda <200\lambda$, D<10λ), the sampling intervals are $\Delta \theta =\Delta \varphi =3^{{}^{\circ}}$, and the radius $r^{\prime \prime }$ of the inversely retrieved reactive near-field sphere $S^{\prime \prime }$ is set as 5.1λ (closely adjacent to the AUT surface). The nine patch elements are simultaneously excited, and the simulated partial-spherical complex near field on $S^{\prime }$ is given in Figure 3, where the truncated spherical near-field range is sampled within $45^{{}^{\circ}}$ (i.e., the reliable region in θ direction is $\pm 45^{{}^{\circ}}$), since the maximum geometrical angle of the conformal AUT in θ direction is $30^{{}^{\circ}}$.

**Figure 4:**
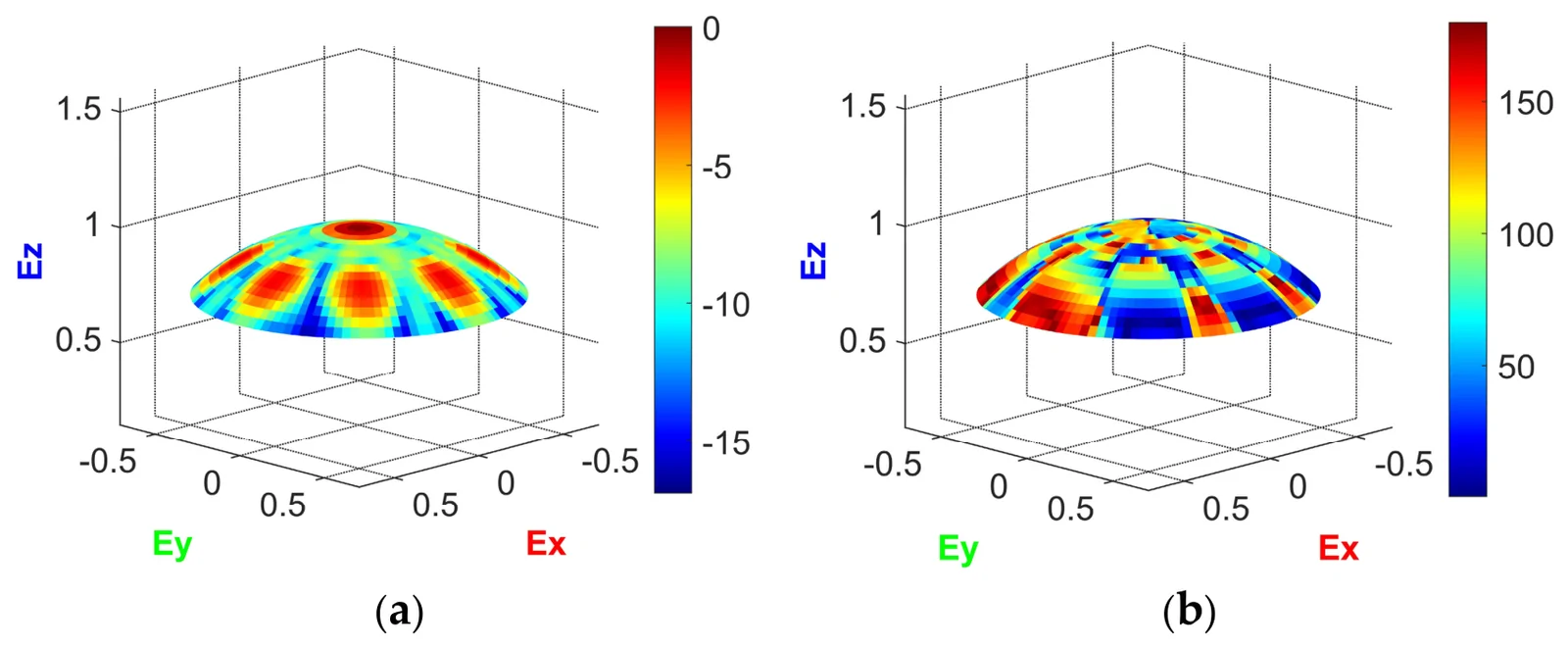
Retrieved simulated reactive near field by the proposed deconvolution method on $S^{\prime \prime }$: (**a**) amplitude and (**b**) phase.

**Figure 5:**
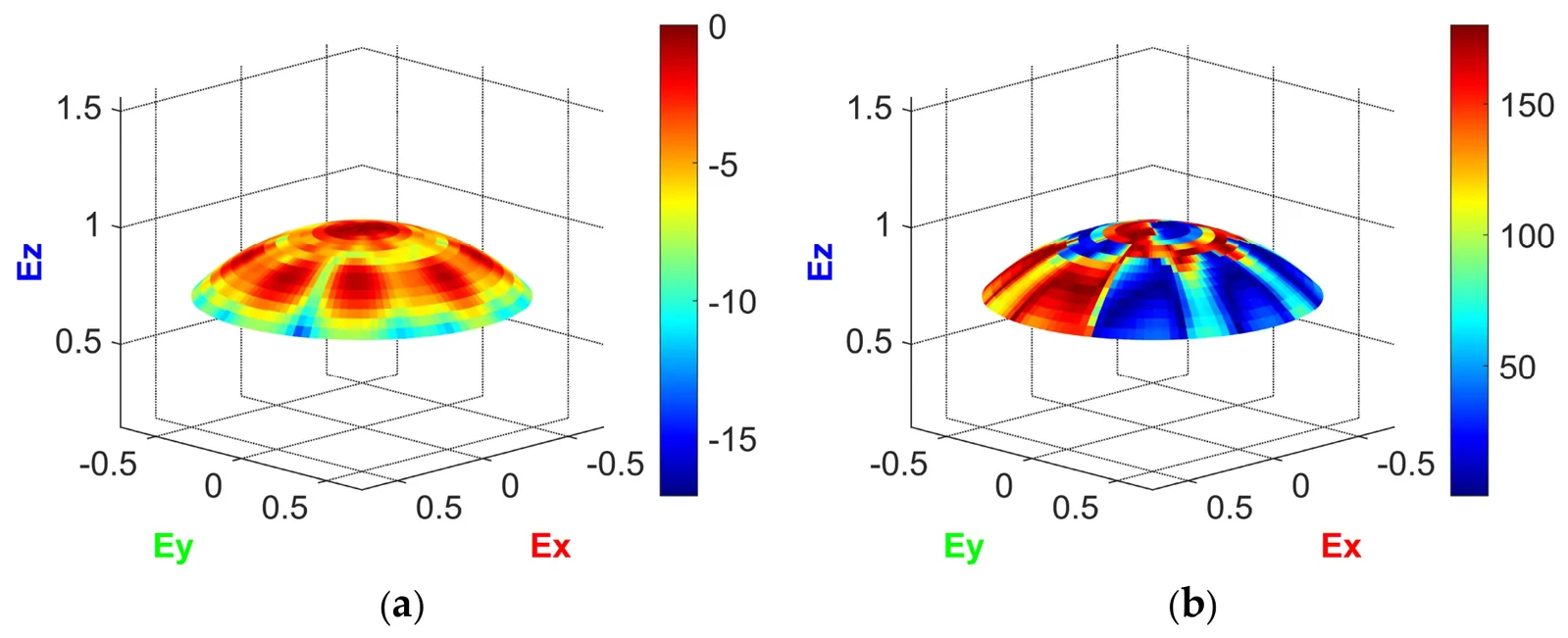
Retrieved simulated reactive near field by the conventional method on $S^{\prime \prime }$: (**a**) amplitude and (**b**) phase.

**Figure 6:**
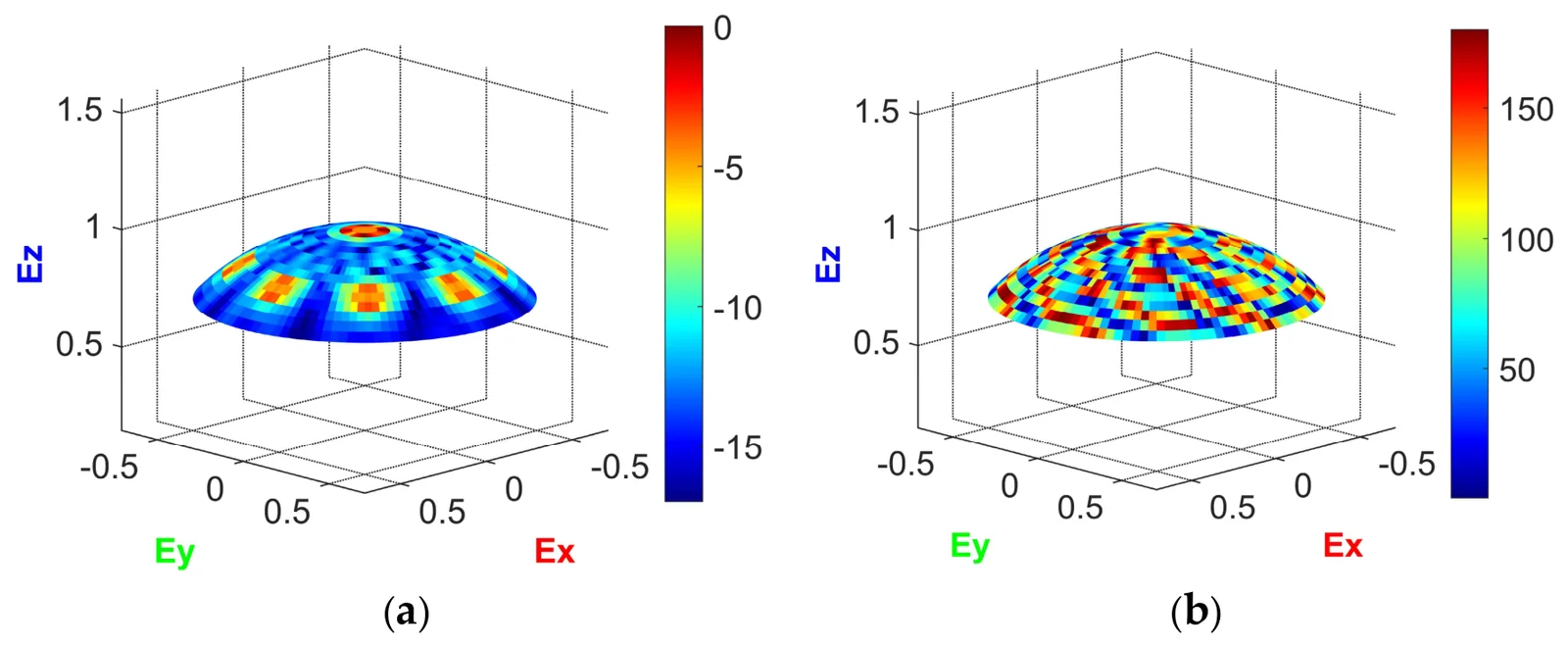
Theoretical reactive near field on $S^{\prime \prime }$ simulated in FEKO: (**a**) amplitude and (**b**) phase.

The deconvolution kernel G is defined as a partial-spherical dome with the ranges of $\theta =(0\sim 45^{{}^{\circ}})$ and $\varphi =(0^{{}^{\circ}}\sim 360^{{}^{\circ}})$, whose moving intervals are $\Delta \theta =\Delta \varphi =3^{{}^{\circ}}$. Figure 4 shows the inversely retrieved reactive complex near-field on $S^{\prime \prime }$ obtained by the proposed deconvolution method. To compare with the proposed method, the conventional spherical-wave expansion-based method [1] is adopted to inversely derive the reactive near field on $S^{\prime \prime }$, whose reactive complex near field is shown in Figure 5. In addition, Figure 6 shows the partial-spherical theoretical reactive near field simulated in FEKO.

**Figure 7:**
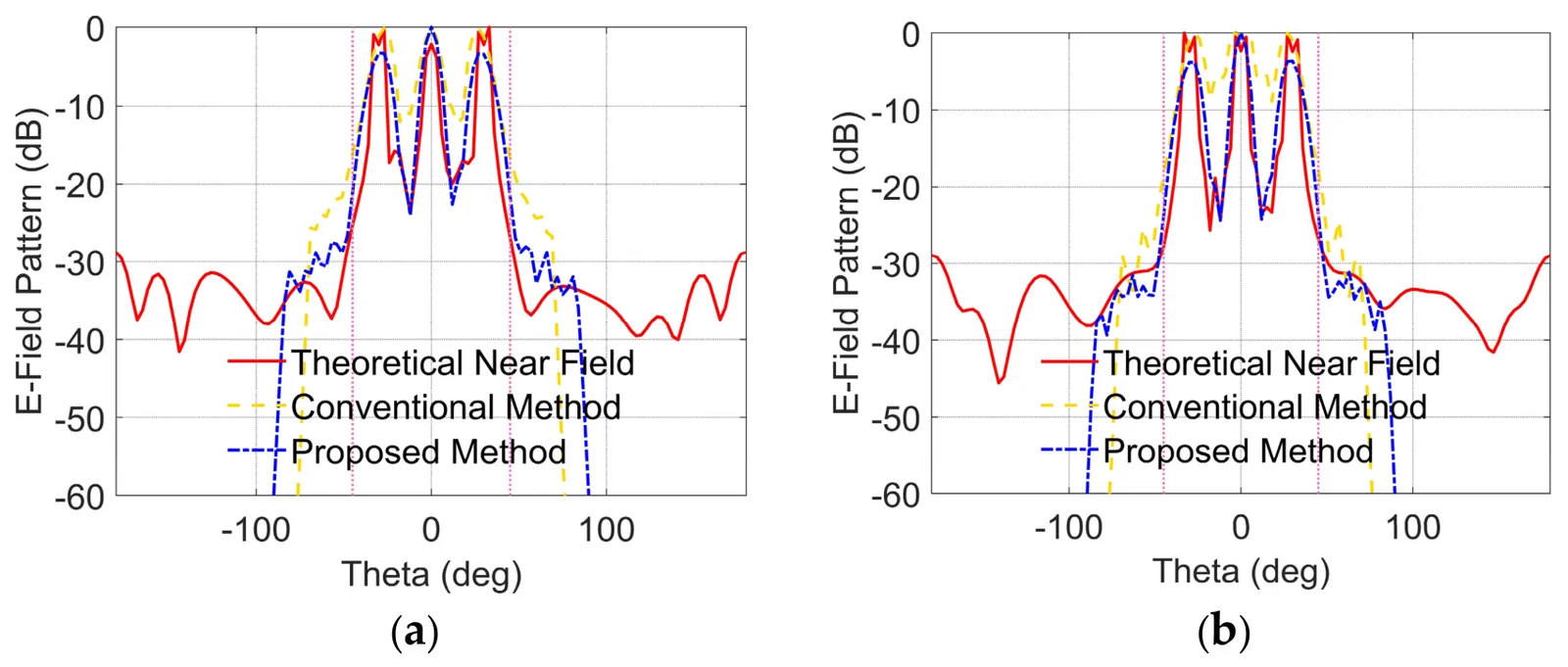
Comparisons of the reactive near field in the cutting plots of (**a**) $\varphi =0^{{}^{\circ}}$ and (**b**) $\varphi =90^{{}^{\circ}}$.

The comparisons in the cutting plots of the reactive near-field amplitudes in Figure 4, Figure 5 and Figure 6 are given in Figure 7, where the red, yellow, and blue curves are the theoretical, conventional method-based, and proposed method-based reactive near fields, and the vertical pink line is the reliable region of $\pm 45^{{}^{\circ}}$ in the θ direction.

It should be noted that the θ-range of the deconvolution reactive near field obtained by the proposed method in Figure 7 (i.e., blue curve) is extended to nearly $\pm 90^{{}^{\circ}}$, which is because the length L of the final deconvolution reactive near field in the θ direction is determined by the length $L_{1}$ of the deconvolution kernel G and the length $L_{2}$ of the measured near field in the θ direction, and L can be calculated as $L=L_{1}+L_{2}-1$. In addition, the reactive near field obtained by the conventional method (i.e., yellow curve) is calculated by the wave-expansion method, and the spatial-domain truncation yields signal extension in the wavenumber domain, which results in fitted outputs outside the reliable region ($\pm 45^{{}^{\circ}}$) for the back-propagated near-field from the spectral domain. Therefore, the theoretical near field (i.e., red curve) within $\theta =\pm 180^{{}^{\circ}}$ is presented to compare with the extrapolation performance of the reconstructed reactive near fields by the two methods. Accordingly, the proposed method can retrieve the complex reactive near field to enable element diagnosis when the amplitude and phase of some elements show apparent attenuation and deviation compared with other elements or the benchmark.

### 3.2. Measurement Results

**Figure 8:**
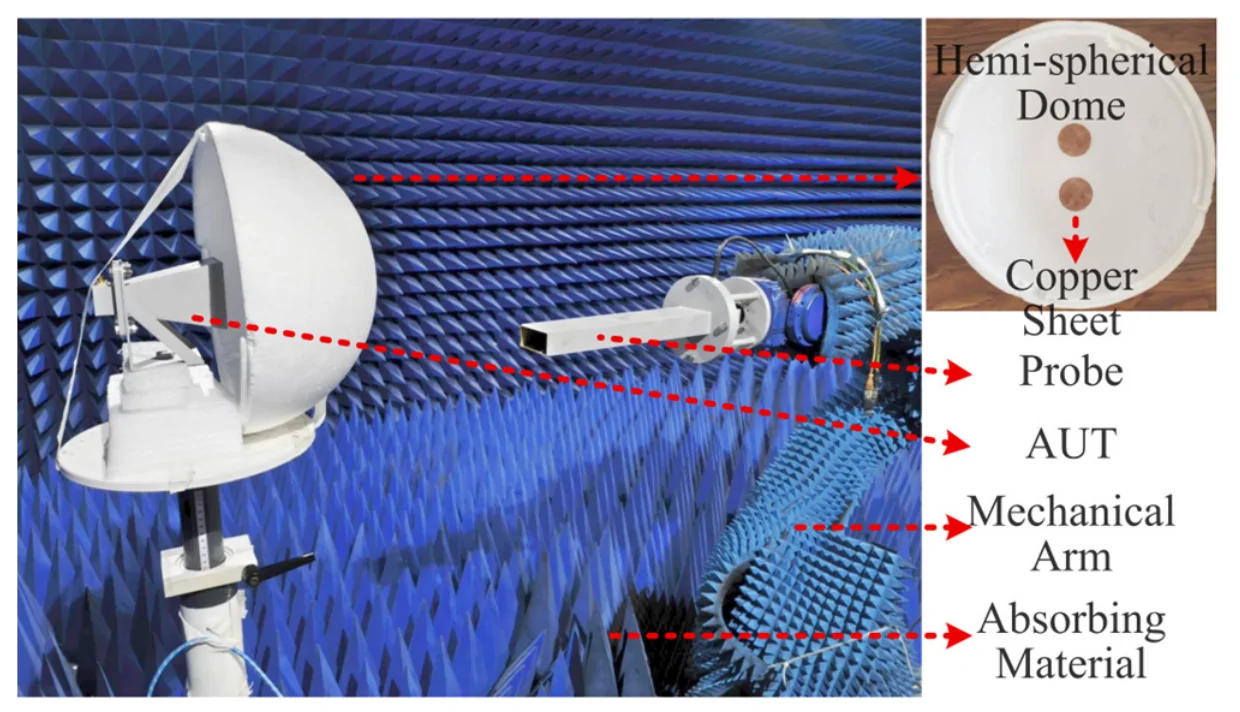
Measurement setup.

**Figure 9:**
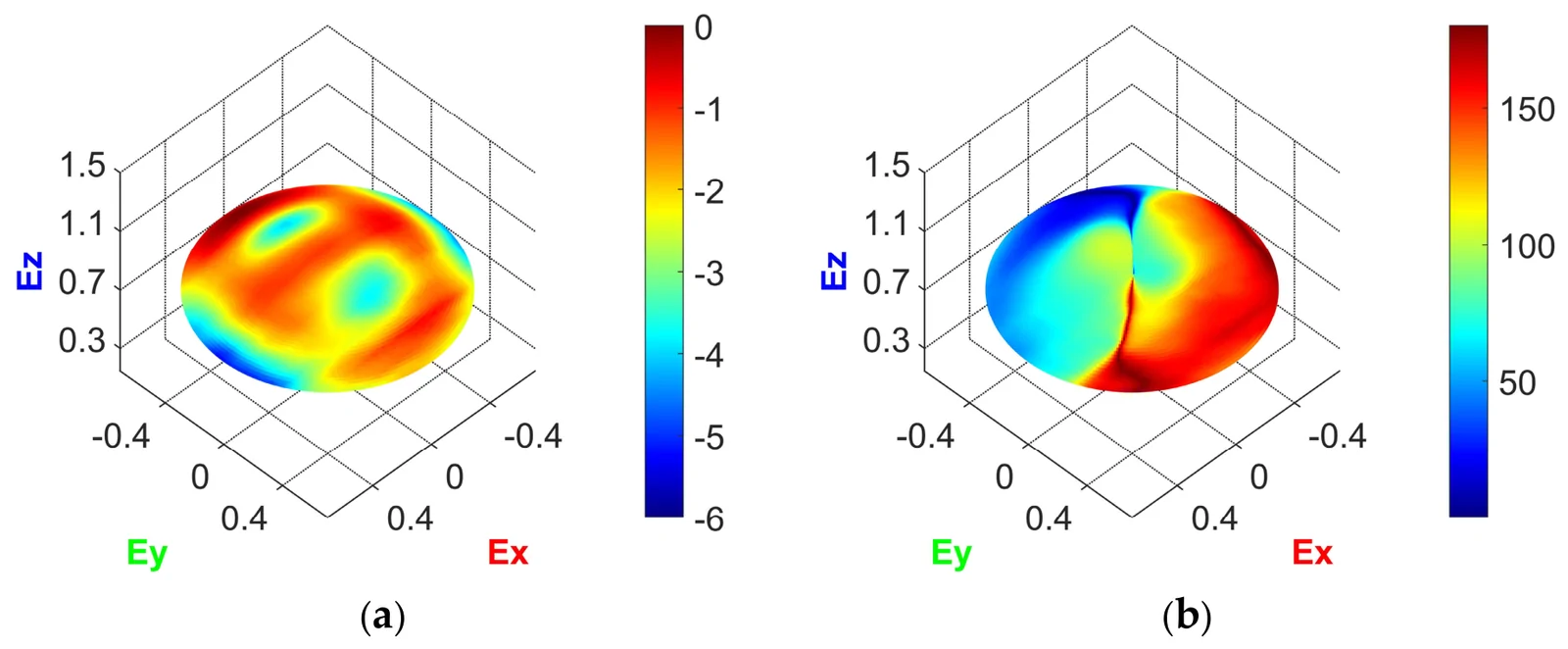
Measured radiative near field on $S^{\prime }$: (**a**) amplitude and (**b**) phase.

Figure 8 shows the partial-spherical near-field measurement, where the AUT is a horn antenna operating at 3 GHz. A hemispherical foam dome with a radius of 2λ is mounted in front of the AUT, where the AUT aperture center coincides with the hemispherical dome center (i.e., the vertical distance between the hemispherical dome surface and the AUT aperture is 2λ). In addition, two round copper sheets with a radius of 0.3λ are fixed on the inner surface of the dome, where the distance between the two copper sheets is 1λ. These copper sheets introduce controllable field diffraction to form a known equivalent source distribution on the inner virtual surface, serving as a benchmark for aperture-field diagnosis. Then, the radiative near field is measured on the partial surface $S^{\prime }$ with a radius of $r^{\prime }=4\lambda$, where the testing ranges in the θ and φ directions are $\theta =(0\sim 45^{{}^{\circ}})$ and $\varphi =(0^{{}^{\circ}}\sim 360^{{}^{\circ}})$, and the sampling intervals are $\Delta \theta =\Delta \varphi =1^{{}^{\circ}}$. This truncated angular range mimics practical measurement constraints where full-sphere scanning is inaccessible. A vector network analyzer is adopted to record the complex amplitude and phase of the near-field signal at each sampling grid, and precise alignment is performed to guarantee the origin consistency of the spherical coordinate system. The measured near field on $S^{\prime }$ is shown in Figure 9, whose reliable range in θ is limited to $\pm 45^{{}^{\circ}}$.

**Figure 10:**
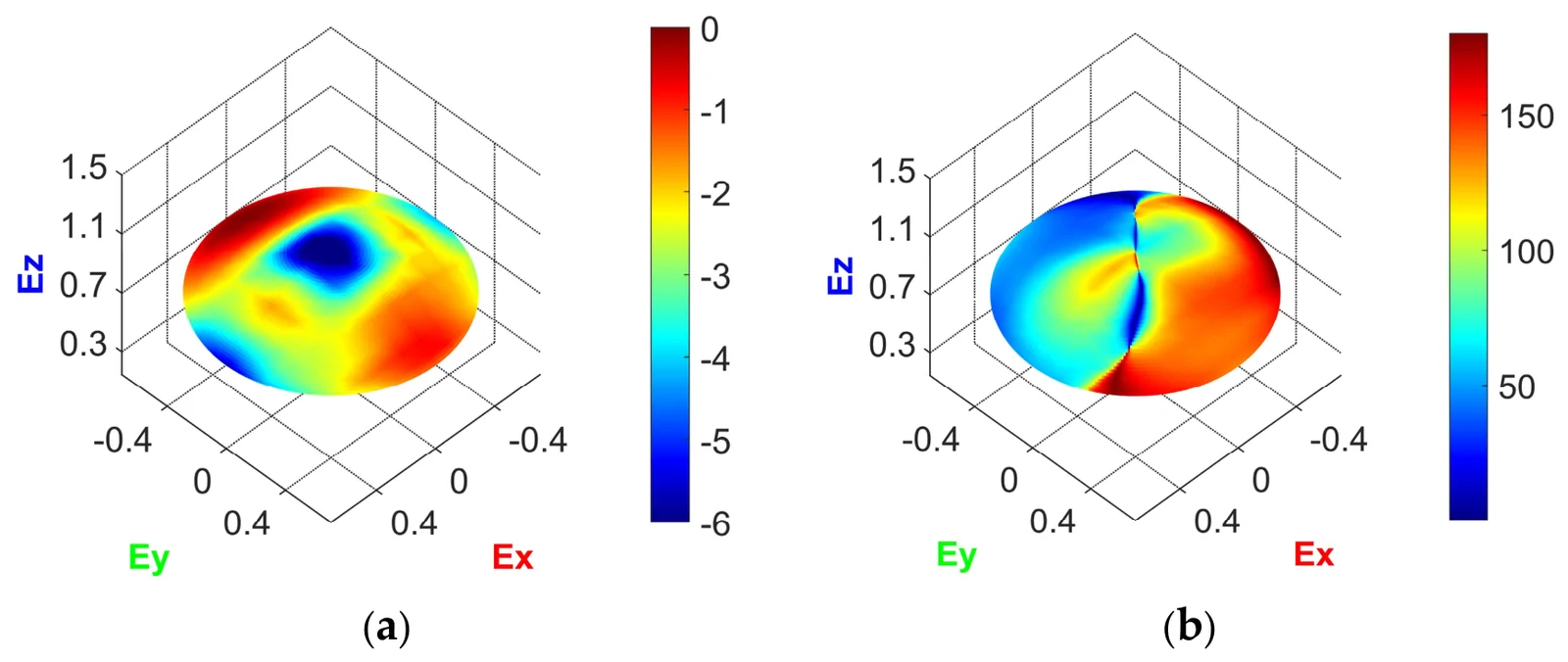
Retrieved measured reactive near field by the proposed deconvolution method on $S^{\prime \prime }$: (**a**) amplitude and (**b**) phase.

**Figure 11:**
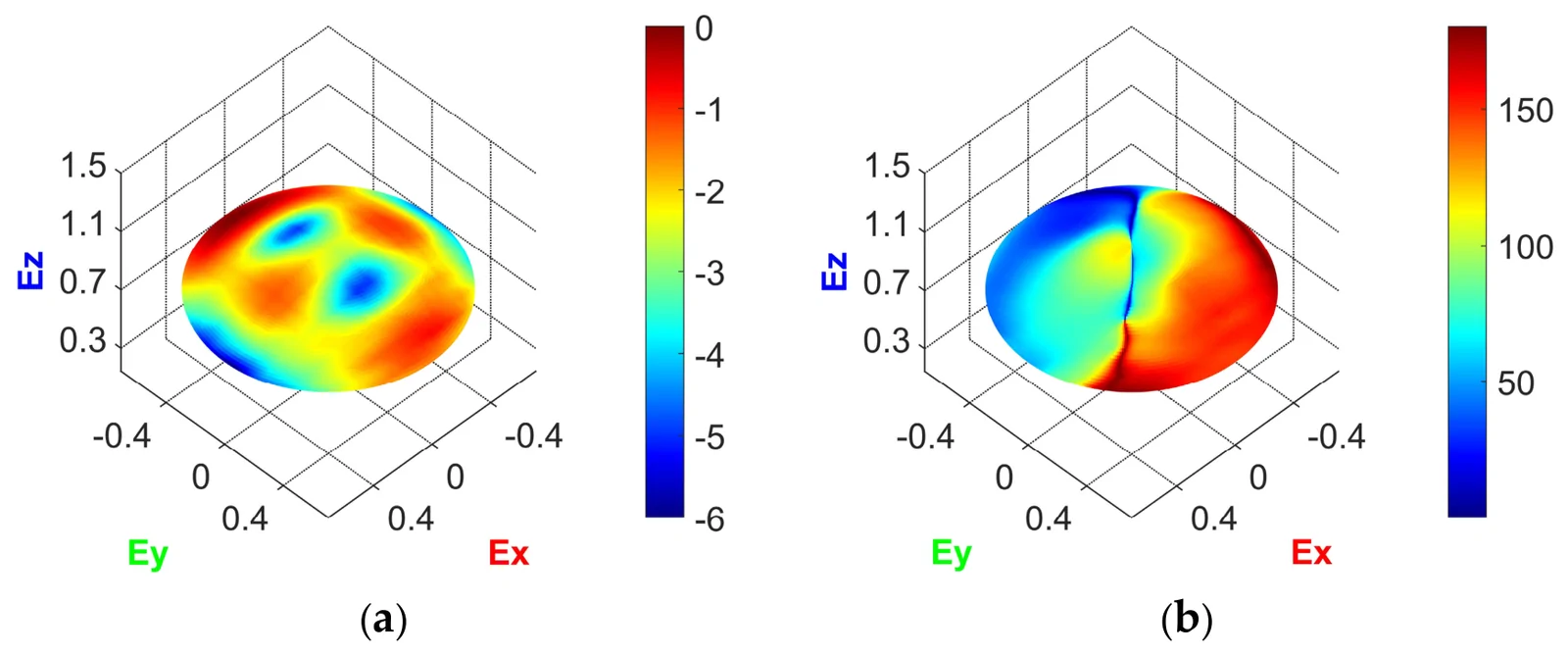
Retrieved measured reactive near field by the conventional method on $S^{\prime \prime }$: (**a**) amplitude and (**b**) phase.

**Figure 12:**
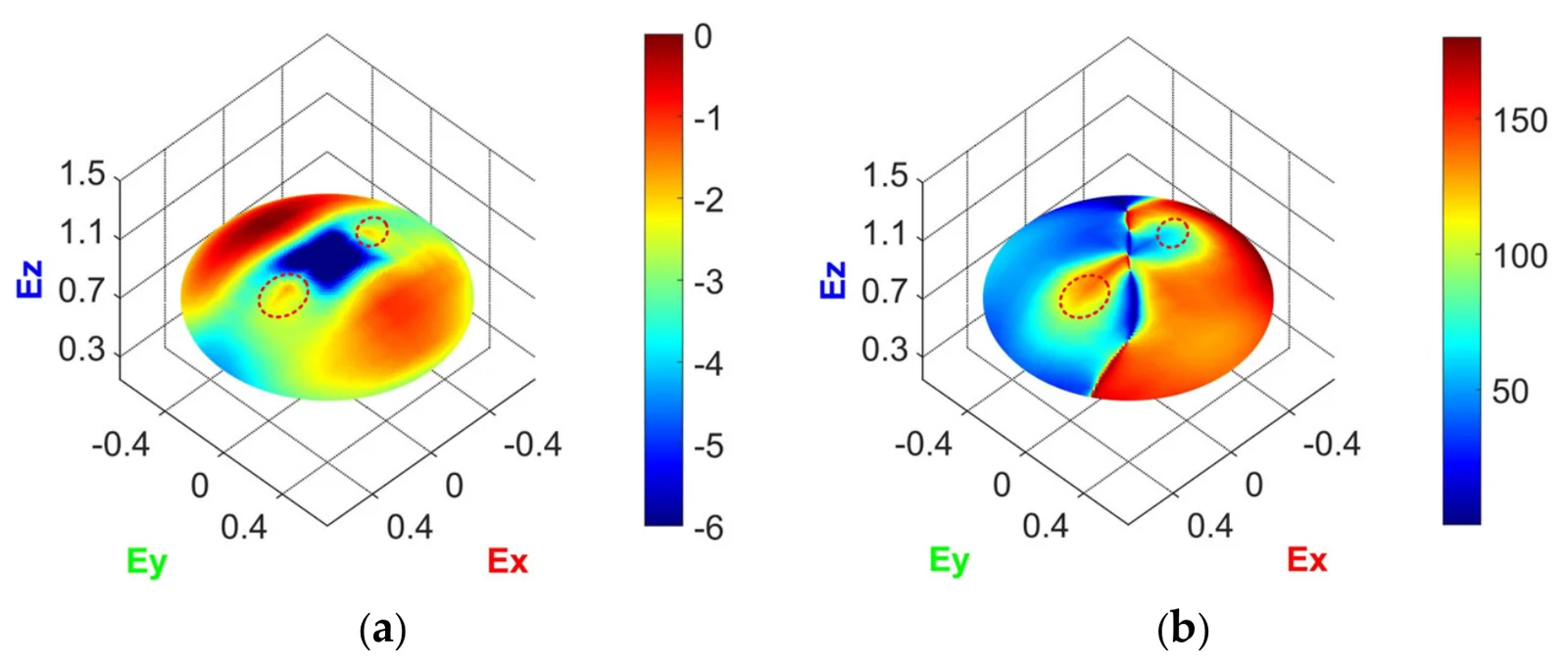
Directly measured reactive near field on $S^{\prime \prime }$: (**a**) amplitude and (**b**) phase.

**Figure 13:**
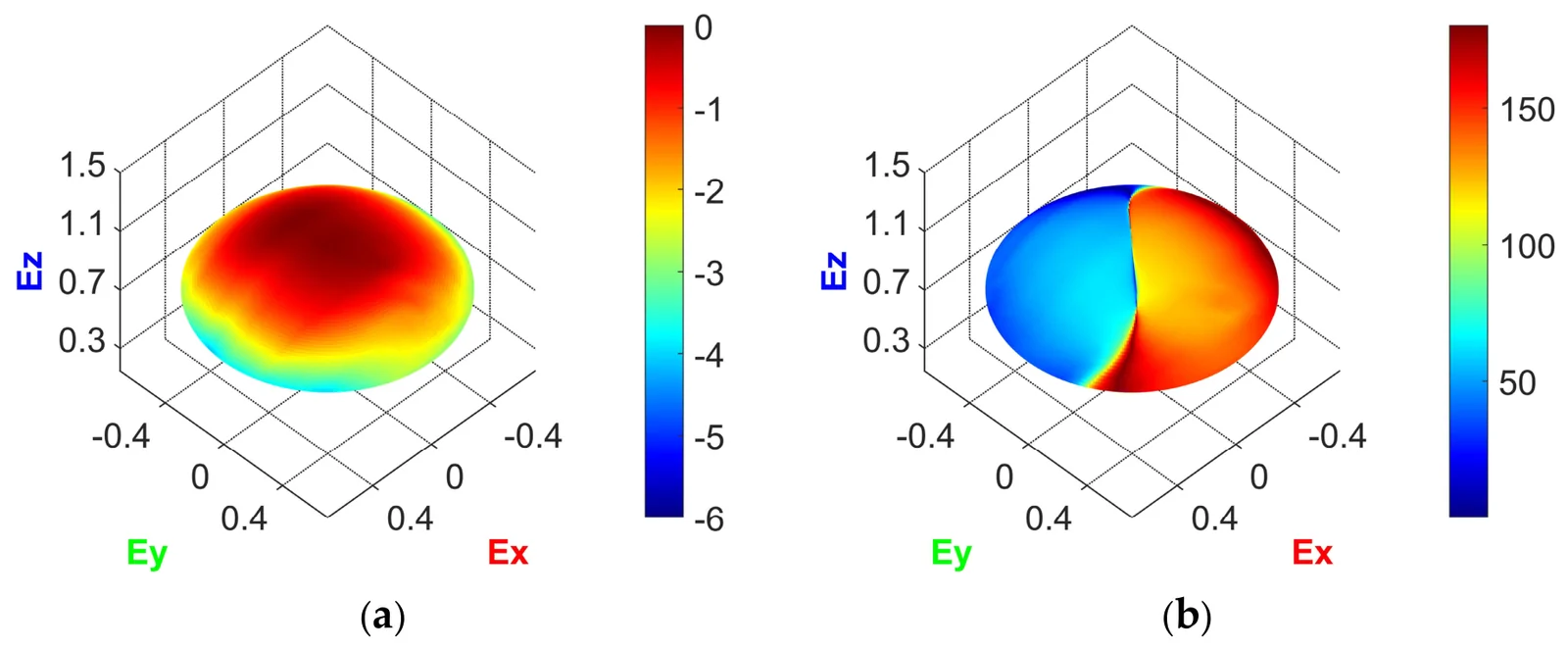
Directly measured reactive near field on $S^{\prime \prime }$ without copper sheets: (**a**) amplitude and (**b**) phase.

The size of G is defined as $\theta =(0\sim 45^{{}^{\circ}})$ and $\varphi =(0^{{}^{\circ}}\sim 360^{{}^{\circ}})$, with the moving intervals of $\Delta \theta =\Delta \varphi =1^{{}^{\circ}}$. The reactive near fields inversely retrieved by the proposed method and the conventional method on *S*″ with a radius of $r^{\prime \prime }=2.1\lambda$ are given in Figure 10 and Figure 11, respectively. The directly measured reactive near field on *S*″ is presented in Figure 12, where the spatial positions corresponding to the diffraction of the two copper sheets are marked with red dashed circles. To clearly demonstrate the variation of the reactive near-field distribution with and without copper sheets, Figure 13 shows the ideal near field on *S*″ when the copper sheets are removed. Therefore, based on the results in Figure 10, Figure 11, Figure 12 and Figure 13, the two copper sheets cause significant reactive near-field changes, including amplitude attenuation and phase mutation.

### 3.3. Discussion

#### 3.3.1. Relative Errors

The relative errors of the comparisons in the cutting plots of $\varphi =0^{{}^{\circ}}$ and $\varphi =90^{{}^{\circ}}$ are calculated as

(20) Er=∑θ=−45°θ=45°ETheor(θ,φ)−ERe(θ,φ)2∑θ=−45°θ=45°ETheor(θ,φ))2

where $E_{\mathrm{Theor}}$ is the theoretical (or referenced) reactive near field, ERe is the retrieved reactive near field by the proposed or conventional methods, and the reliable region is $\theta =\pm 45^{{}^{\circ}}$. The simulated relative errors of Figure 7 are summarized in Table 1.

**Figure 14:**
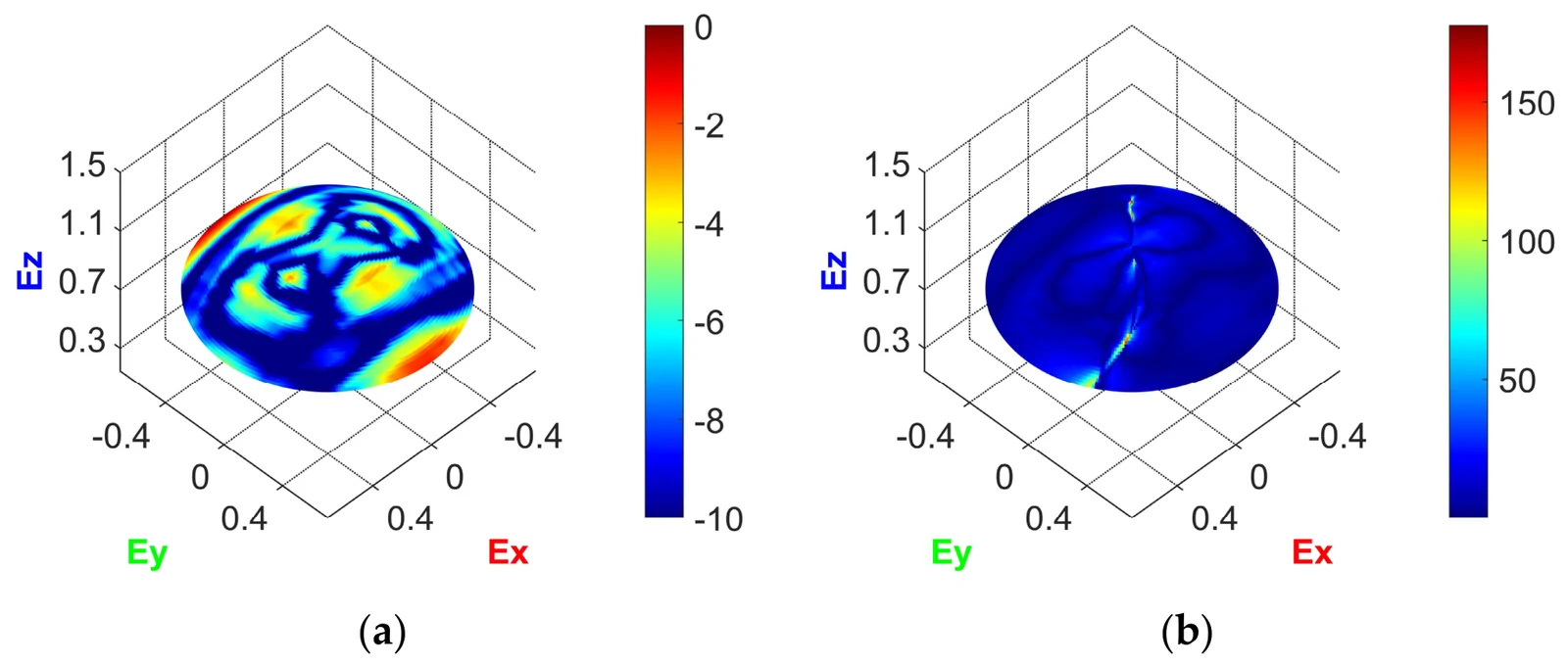
Errors between the near field directly measured and the near field retrieved by the proposed method: (**a**) amplitude and (**b**) phase.

**Figure 15:**
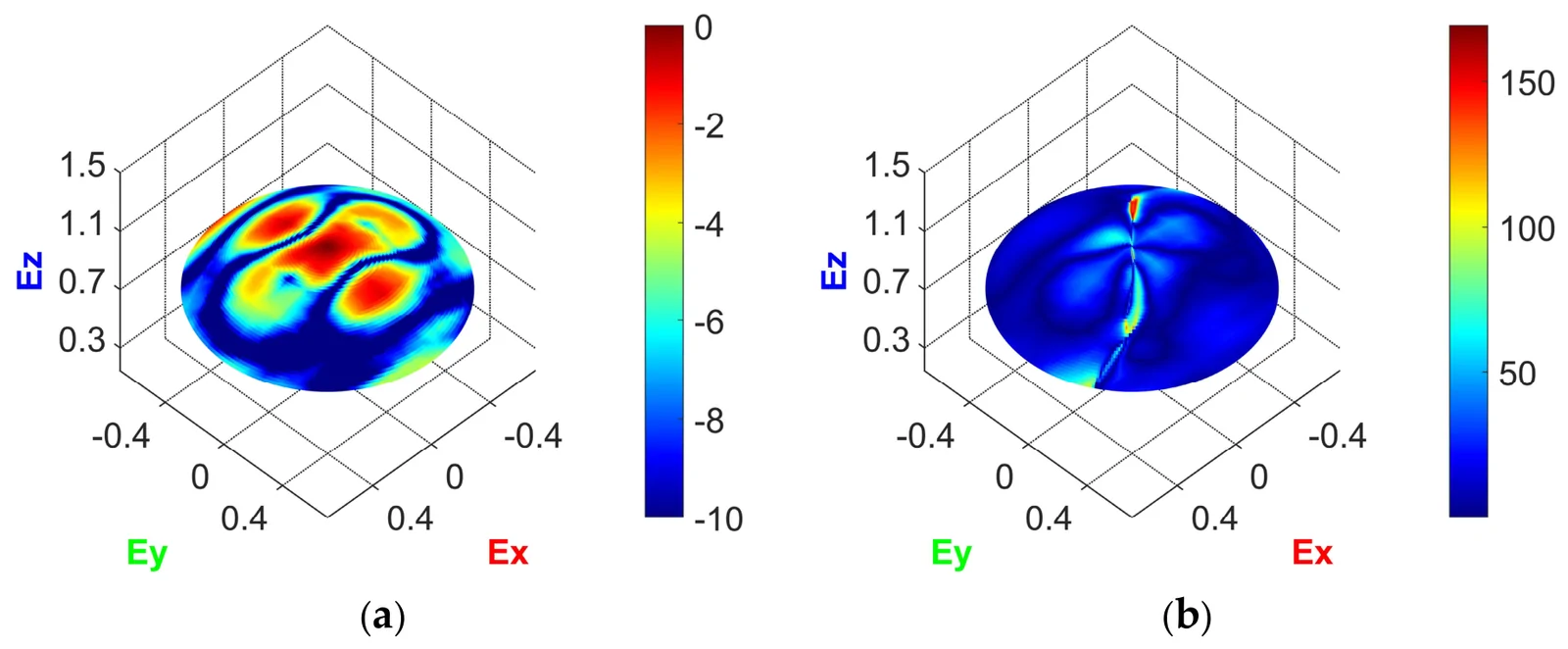
Errors between the near field directly measured and the near field retrieved by the conventional method: (**a**) amplitude and (**b**) phase.

In addition, Figure 14 and Figure 15 show the partial-spherical errors between the measured near field (Figure 12) and the near fields inversely retrieved by the proposed and conventional methods (Figure 10 and Figure 11), respectively. The corresponding relative errors can be calculated along all the cutting planes as

(21) Er=∑φ=0°φ=360°∑θ=0°θ=45°EMea(θ,φ)−ERe(θ,φ)2∑φ=0°φ=360°∑θ=0°θ=45°EMea(θ,φ)2

where $E_{\mathrm{Mea}}$ is the measured near field in Figure 12, and ERe is the retrieved near field by the two methods in Figure 10 and Figure 11. The measured relative errors of Figure 14 and Figure 15 are summarized in Table 2, and it is clear that the proposed method can effectively derive the reactive near field and is more accurate than the conventional method.

#### 3.3.2. Far-Field Retrieving Performance

**Figure 16:**
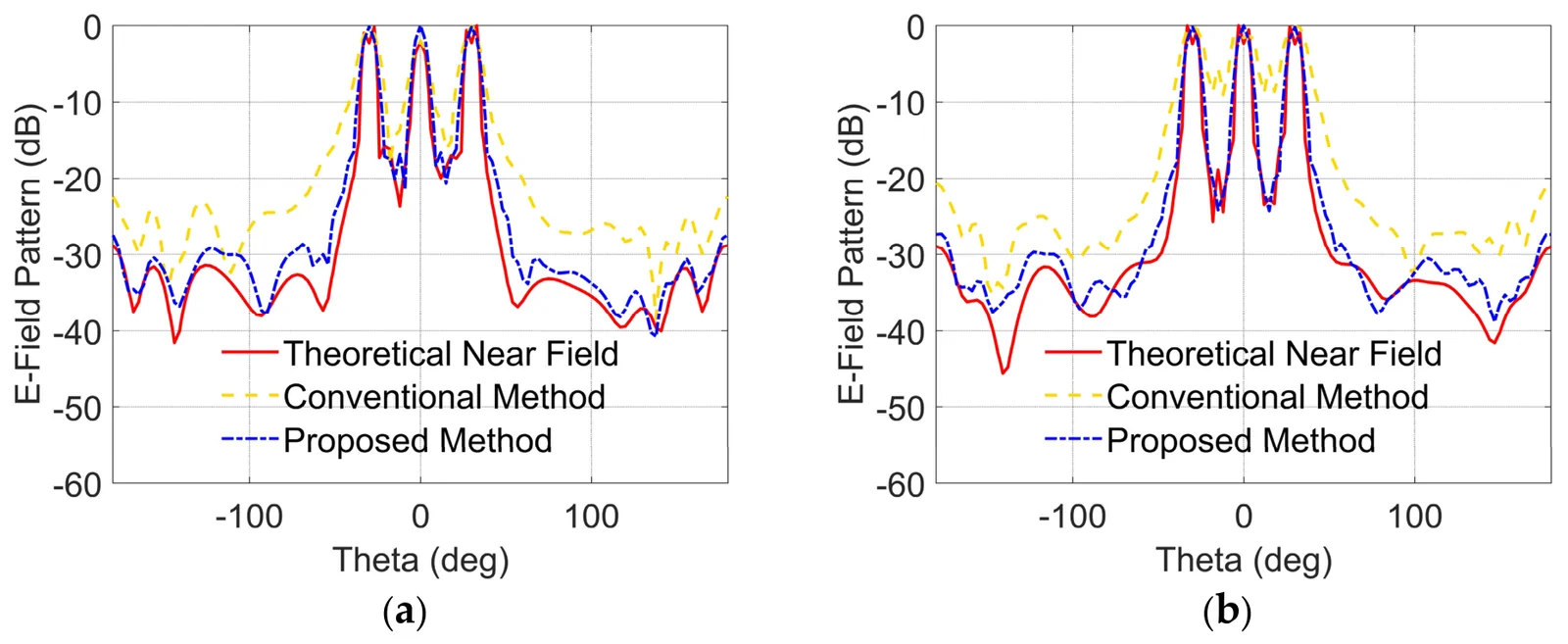
Comparisons of the reactive near field retrieved from the far-field pattern in the cutting plots of (**a**) $\varphi =0^{{}^{\circ}}$ and (**b**) $\varphi =90^{{}^{\circ}}$.

The results above show the proposed method’s effectiveness for the radiative near field. To further verify the proposed method for retrieving the reactive near field from the full-sphere far-field pattern, the following presents the corresponding simulation results of the same conformal AUT shown in Figure 2. The far-field radius $r^{\prime }$ of the measured sphere $S^{\prime }$ is set as 200λ; the full-sphere sampling ranges are $\theta =(0\sim 180^{{}^{\circ}})$ and $\varphi =(0^{{}^{\circ}}\sim 360^{{}^{\circ}})$ with sampling intervals of $\Delta \theta =\Delta \varphi =1^{{}^{\circ}}$. The radius $r^{\prime \prime }$ of the retrieved near-field sphere $S^{\prime \prime }$ is 5.1λ, and the scanning ranges of the partial-spherical deconvolution kernel G are redefined as $\theta =(0\sim 60^{{}^{\circ}})$ and $\varphi =(0^{{}^{\circ}}\sim 360^{{}^{\circ}})$ with the moving interval of $1^{{}^{\circ}}$. Then, the cutting plots of the retrieved near field from the far-field pattern in $\varphi =0^{{}^{\circ}}$ and $\varphi =90^{{}^{\circ}}$ are shown in Figure 16, whose relative errors are calculated by Equation (20) with the reliable region of $\theta =\pm 180^{{}^{\circ}}$ and summarized in Table 3.

Based on the simulation and measurement results shown above, the proposed method effectively retrieves the reactive near field from the measured near field or far field.

#### 3.3.3. Application Advantage

**Figure 17:**
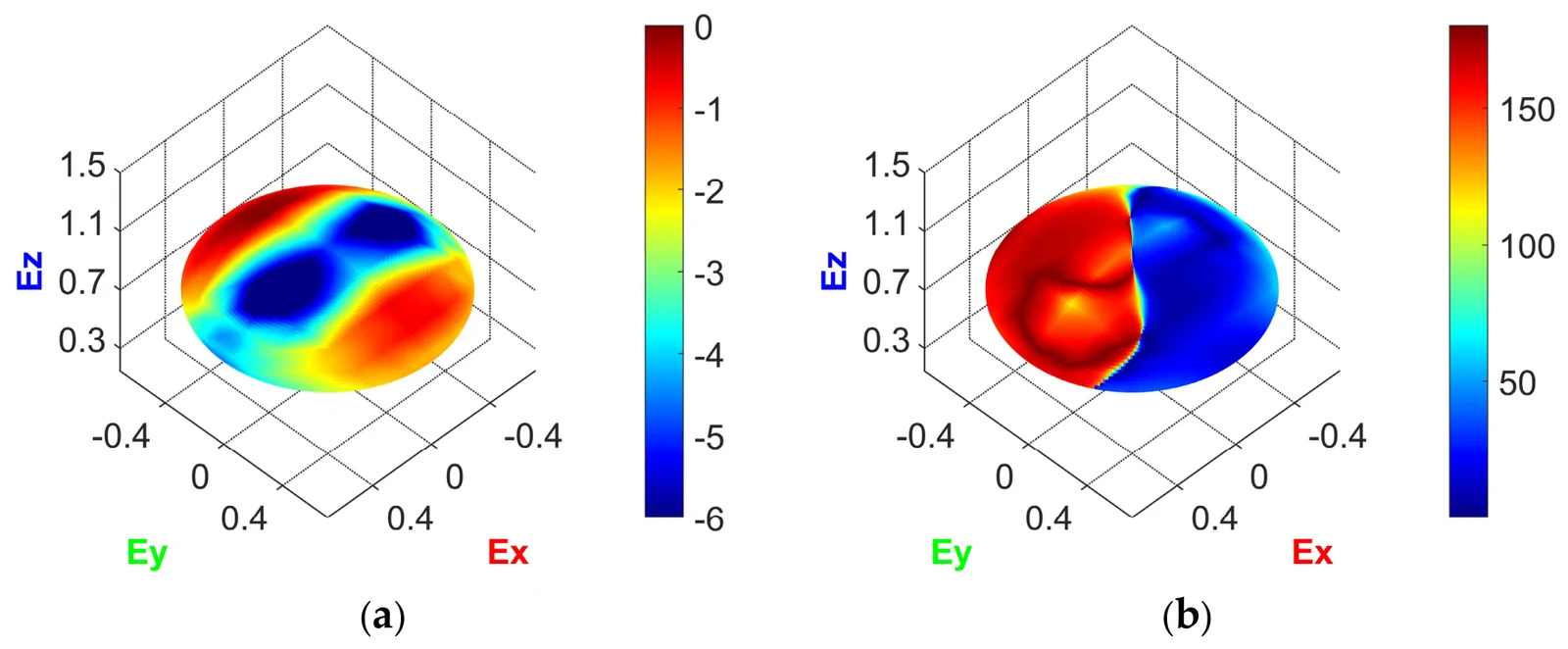
Retrieved measured reactive near field on the inner surface of the dome by the proposed deconvolution method on $S^{\prime \prime }$: (**a**) amplitude (**b**) phase.

The directly measured near field can only be obtained within a range of $r^{\prime \prime }=2.1\lambda$ meters or more; in other words, the reactive near field on the inner virtual surface of the dome cannot be directly acquired. Thus, to reveal the aperture field characteristics, the accessible way is to use the proposed method to retrieve the reactive near field with a radius less than 2λ. In this regard, the retrieved reactive near field on $S^{\prime \prime }$ with a radius of $r^{\prime \prime }=1.3\lambda$ is shown in Figure 17. The amplitude attenuation and phase variation correspond to diffraction by the two copper sheets, which can be modeled as an equivalent source distribution on the inner virtual surface $S^{\prime \prime }$. Therefore, the proposed method can be widely used in scenarios where direct internal measurement is not possible, such as hermetically packaged millimeter-wave transceivers, aircraft conformal antenna arrays protected by radomes, and compact integrated on-chip antennas, enabling element-level fault diagnosis, equivalent source modeling and internal reactive near-field characterization without probe intrusion.

#### 3.3.4. Computational Cost

The time cost for the single cutting plane at $\varphi =0^{{}^{\circ}}$ or $\varphi =90^{{}^{\circ}}$ in Figure 7 is 18 min, where the scanning interval of the spatial deconvolution kernel *G* is $3^{{}^{\circ}}$. Meanwhile, the time cost for the single cutting plane $\varphi =0^{{}^{\circ}}$ or $\varphi =90^{{}^{\circ}}$ in Figure 16 is 5 h, where the scanning interval of the spatial deconvolution kernel *G* is $1^{{}^{\circ}}$. Both programs are executed on a personal computer with a 2.6 GHz Core i9-13900H CPU and 32 GB RAM. In contrast, the conventional spherical-wave expansion-based method is 3 min and 1 h for the single cuts in Figure 7 and Figure 16, respectively. Although the conventional method has a lower time cost, the better accuracy of the proposed method is more important in element diagnosis.

Therefore, the larger the deconvolution kernel (i.e., the smaller the scanning interval), the higher the time cost. For the same AUT model, a smaller scanning interval (still larger than the spherical Nyquist sampling interval) yields a more accurate reactive near field. Consequently, trade-offs between time cost and method accuracy can be made according to specific testing requirements.

#### 3.3.5. Experimental Uncertainties Discussion

(1) Antenna mismatch

Antenna mismatch introduces multiple reflections along the feed chain, which distorts the absolute field amplitude and phase offset. For ill-posed inverse source reconstruction and under-sampling recovery in [8,9,10,11], phase errors from mismatch break the coherence of the sampled field data. This increases the reconstruction error and produces faulty sources in the inverted result. While the proposed method is not influenced by the inverse problem, and the phase error only slightly impacts the normalized reconstruction accuracy, for future quantitative measurements and improvements, the S-parameters will be acquired for mismatch correction.

(2) Probe positioning

Deviations from the targeted spherical surface introduce position-dependent phase errors. The radial offset directly changes the propagation distance and phase term. In this work, the testing frequency is 3 GHz, and the small positioning jitter is relatively smaller than the Nyquist sampling interval, which brings moderate phase perturbation and does not break the global coherence required by the deconvolution method. However, larger positioning errors would produce noticeable phase errors and increase the inaccuracy of the reconstructed source distribution.

(3) Calibration

The amplitude–phase calibration of the vector network analyzer channel is performed prior to the measurement, which removes the cable loss, phase offset and receiver drift. After the probe positioning and the phase center determination, the far-field pattern offset can be calibrated by removing the global coordinate system, and the far field accuracy can be improved [25].

(4) Spatial resolution

The spatial resolution of the proposed deconvolution method is mainly determined by the size of the deconvolution kernel G and the measured spherical electric field. On the one hand, since the size and scanning interval of G can be arbitrarily set, the accuracy of the deconvoluted reactive near field can be improved by the enlargement of G, where more effective spherical data can be enclosed and used to calculate the inner reactive near field. On the other hand, when the sampling interval of the measured electric field on $S^{\prime }$ is reduced to less than the Nyquist sampling theorem, the deconvoluted near field can be naturally more accurate.

## 4. Conclusions

This paper presents and verifies an effective reactive near-field retrieval method. The proposed method uses spherical integrals and element-wise matrix division to perform spherical deconvolution in the wavenumber domain, and its computational overhead depends on the deconvolution kernel size. Both simulation and measurement results demonstrate the proposed method’s accuracy compared to the spherical wave expansion-based conventional method. More importantly, the trade-offs between the time cost and accuracy can be adjusted flexibly to meet specific engineering requirements to achieve optimal application goals.

## Figures and Tables

**Table 1:** Simulated near-field retrieved relative errors.

Relative Errors	Proposed Method	Conventional Method	Improvements
$\varphi =0^{{}^{\circ}}$	8.64%	14.17%	5.53%
$\varphi =90^{{}^{\circ}}$	8.07%	17.29%	9.22%

**Table 2:** Measured near-field retrieved relative errors.

Relative Errors	Proposed Method	Conventional Method	Improvements
Amplitude	12.06%	33.61%	21.55%
Phase	11.45%	21.42%	9.97%

**Table 3:** Simulated far-field retrieved relative errors.

Relative Errors	Proposed Method	Conventional Method	Improvements
$\varphi =0^{{}^{\circ}}$	2.51%	8.31%	5.80%
$\varphi =90^{{}^{\circ}}$	2.28%	9.63%	7.35%

## Data Availability

All data are available from the author.
